# Multifactorial Likelihood Assessment of *BRCA1* and *BRCA2* Missense Variants Confirms That *BRCA1*:c.122A>G(p.His41Arg) Is a Pathogenic Mutation

**DOI:** 10.1371/journal.pone.0086836

**Published:** 2014-01-28

**Authors:** Phillip J. Whiley, Michael T. Parsons, Jennifer Leary, Kathy Tucker, Linda Warwick, Belinda Dopita, Heather Thorne, Sunil R. Lakhani, David E. Goldgar, Melissa A. Brown, Amanda B. Spurdle

**Affiliations:** 1 QIMR Berghofer Medical Research Institute, Brisbane, Queensland, Australia; 2 School of Chemistry and Molecular Biosciences, University of Queensland, Brisbane, Queensland, Australia; 3 Familial Cancer Service, Westmead Institute for Cancer Research, University of Sydney at Westmead Millennium Institute, Westmead Hospital, Sydney, NSW, Australia; 4 Hereditary Cancer Clinic, Prince of Wales Hospital, Randwick, Sydney, NSW, Australia; 5 Genetics Department, The Canberra Hospital, Canberra, ACT, Australia; 6 kConFab, Peter MacCallum Cancer Centre, Melbourne, Victoria, Australia; 7 The University of Queensland, UQ Centre for Clinical Research and School of Medicine, and Pathology Queensland, The Royal Brisbane and Women’s Hospital, Brisbane, Queensland, Australia; 8 Huntsman Cancer Institute and Department of Dermatology, University of Utah, Salt Lake City, Utah, United States of America; IFOM, Fondazione Istituto FIRC di Oncologia Molecolare, Italy

## Abstract

Rare exonic, non-truncating variants in known cancer susceptibility genes such as *BRCA1* and *BRCA2* are problematic for genetic counseling and clinical management of relevant families. This study used multifactorial likelihood analysis and/or bioinformatically-directed mRNA assays to assess pathogenicity of 19 *BRCA1* or *BRCA2* variants identified following patient referral to clinical genetic services. Two variants were considered to be pathogenic (Class 5). *BRCA1*:c.4484G> C(p.Arg1495Thr) was shown to result in aberrant mRNA transcripts predicted to encode truncated proteins. The *BRCA1*:c.122A>G(p.His41Arg) RING-domain variant was found from multifactorial likelihood analysis to have a posterior probability of pathogenicity of 0.995, a result consistent with existing protein functional assay data indicating lost BARD1 binding and ubiquitin ligase activity. Of the remaining variants, seven were determined to be not clinically significant (Class 1), nine were likely not pathogenic (Class 2), and one was uncertain (Class 3).These results have implications for genetic counseling and medical management of families carrying these specific variants. They also provide additional multifactorial likelihood variant classifications as reference to evaluate the sensitivity and specificity of bioinformatic prediction tools and/or functional assay data in future studies.

## Introduction

Identification of missense *BRCA1* and *BRCA2* unclassified variants during clinical testing poses a problem for clinicians and affected families, given their unclear role in disease risk and tumorigenesis. The multifactorial likelihood model for variant classification has been proposed as a gold standard for variant classification. The method utilizes statistical methods incorporating a prior probability of pathogenicity based on bioinformatic predictions, combined with clinical data from tumor pathology, segregation of the variant with disease, family history and co-occurrence with a deleterious mutation data to assign clinical significance [1], [2]. The model derives a posterior probability of pathogenicity for individual variants, and this posterior probability was used as the basis for a 5-tier classification system with associated clinical recommendations [3].

Refinement of the model is an ongoing process with the potential to improve its accuracy through the inclusion of new findings. These may include results that improve the bioinformatically-based estimation of prior probability of pathogenicity, impact the underlying assumptions for estimation of likelihood ratios, revise existing likelihood ratios based on analysis of larger sample sets, and/or estimate likelihood ratios for new components of the model that represent independent data sources.

For instance, the prior probability for an exonic variant is currently based on bioinformatic prediction of the consequences of the amino acid change and does not take into account the potential for a splicing aberration – an important consideration particularly for exonic variants that occur near to the intron-exon boundary or silent variants predicted to create splicing aberrations [4]. It is now feasible to bioinformatically predict whether such variants create *de novo* splice sites with reasonable confidence [5], [6], and rigorous calibration of such predictions against *in vitro* or clinical data will ultimately allow such information to be incorporated into estimates of the prior probability of pathogenicity to improve prediction of pathogenicity for missense variants. As another example, a recent report describing an ovarian cancer patient carrying a pathogenic missense mutation co-occurring *in trans* with a truncating mutation in *BRCA1* [7] indicates inheritance of two pathogenic *BRCA1* mutations may not be lethal as first assumed [8], and that the likelihood ratio developed for co-occurrence of *BRCA1* variants should be amended to reflect this.

Recent studies have assessed the sensitivity and specificity of BRCA1 BRCT domain and BRCA2 DNA-binding domain functional assays to reflect pathogenicity of variants in these domains [9]–[11]. These studies compared functional assay results to pathogenicity assigned on the basis of clinical data alone, and have established the baseline to incorporate data from these specific functional assays into the multifactorial likelihood model. It is acknowledged that other domains of BRCA1 and BRCA2 are important for function, including the RING, transcriptional activation and BRCA1 c-terminal domains. However, to date, there have been no comprehensive studies calibrating level of function of variants in these domains against clinical information, to assess sensitivity and specificity of relevant assays to indirectly measure cancer risk. In addition, the development and calibration of quantitative splicing assays against direct measures of risk will be important to drive improvements in bioinformatic prediction tools, enhance estimation of bioinformatically-determined prior probabilities, and allow incorporation of mRNA assay data as a likelihood component of the multifactorial model.

In this study, we report the results from multifactorial likelihood modeling and/or bioinformatically-directed splicing assays for 19 *BRCA1* and *BRCA2* exonic variants to provide variant classifications of direct clinical utility. The combined bioinformatic, splicing and multifactorial likelihood results contribute to the pool of variants with appropriate clinical classification and assay data that can be used as a calibration set of variants for future studies updating the bioinformatically estimated prior probability of pathogenicity for variants, and also incorporating splicing and functional assays into the multifactorial model.

## Materials and Methods

### Ethics Statement

All families were ascertained as eligible for research by kConFab (http://www.kconfab.org/Index.shtml) [12], apart from two families for which the proband was identified directly by clinical testing in Familial Cancer Clinics. *BRCA1*:c.4484G>C(p.Arg1495Thr) was identified by *BRCA1* mutation screening and referred to the Genetics Department of the Canberra Hospital, Canberra, Australia. Another family carrying the *BRCA1*:c.122A>G(p.His41Arg) variant was recruited by the Familial Service, Westmead Hospital, Westmead, NSW, Australia. *De novo* lymphoblastoid cell lines (LCLs) were established for this study by kConFab, with approval by the Peter Mac Institutional Review Board. Written, informed consent was obtained for all patient samples used and approval was gained from the QIMR Berghofer Human Research Ethics Committee and the Peter Mac Human Research Ethics Committee. All research was conducted in Australia.

Nucleotide numbering reflects cDNA numbering with +1 corresponding to the A of the ATG translation initiation codon in the reference sequence of BRCA1 (GenBank accession #NM_007294.3) and BRCA2 (GenBank accession #NM_000059.3). All 19 exonic variants investigated were considered to be of uncertain clinical significance by the kConFab mutation review committee, or by the investigators (*BRCA1*:c.4484G>C(p.Arg1495Thr)) at the time of selection for the study.

### Bioinformatic Splice Predictions

For all 19 variants investigated, three bioinformatic splice prediction programs (HSF matrices, MaxEntScan and NNsplice) were used to predict whether *de novo* splice sites may be created by variants or whether the variant has an effect on the intron-exon boundary. One program (ESEfinder) was used to assess the effect of a variant on potential exonic splice enhancers. Human Splicing Finder version 2.4 (www.umd.be/HSF/) combines HSF matrices, MaxEnt Scan and ESEfinder in one web interface [13]–[15], and variant nomenclature was input into HSF as in standard HGVS format. Sequences of 25 nucleotides flanking each side of the variant were entered into NNsplice (http://www.fruitfly.org/seq_tools/splice.html) [16]). The difference between variant and wild-type output scores was expressed as a proportion of wild-type scores for HSF matrices and MaxEntScan. Scores for the proximal consensus splice site for all programs were derived by entering the exact sequence at the intron-exon boundary. mRNA assays were prioritized for all variants with existing LCLs, and also for two additional variants where bioinformatic prediction suggested that mRNA splicing might be altered by the variant, namely *BRCA1*:c.4484G>C(p.Arg1495Thr) and *BRCA2* c.7828G>A (p.Val2610Met) (Table 1).

**Table 1:** Bioinformatic splice prediction scores* and *in-vitro* splicing assay results.

Variant		Human Splicing Finder		MaxEntScan		NNsplice		ESEfinder	*In-vitro* splicing assay result
		*Variant*	*Proximal* *consensus site*	*Variant*	*Proximal consensus site*	*Variant*	*Proximal* *consensus site*		
***BRCA1***	c.4484G>C p.Arg1495Thr)	Donor 85.7 (−11.4%)	96.71	Donor 7.69 (−27.2%)	10.57	Donor 0.97	1.00	No enhancer motif	Δexon 14 and Δexon 14/15
	c.4991T>C(p.Leu1664Pro)	Acceptor 71.97 (−2.5%)	87.03	NSC	6.69	NSC	0.61	SF2/ASF: 71.23 (+11.40%)	no aberration
***BRCA2***	c.440A>G(p.Gln147Arg)	Donor 64.23 (−1.24%)	88.86	NSC	9.46	NSC	0.99	SC35: new site SRp40: site broken	no aberration
	c.1514T>C (p.Ile505Thr)	Acceptor 85.55 (+0.49%)	85.7	NSC	9.62	NSC	0.90	No enhancer motif	no aberration
	c.7521A>G (p. = )	Acceptor 75.63 (−0.09%)	82.1	Donor 6.52 (+3.82%)	6.97	NSC	0.90	SF2/ASF (IgM-BRCA1): 88.38 (+20.16%) SF2/ASF: 88.53 (+20.35%)	no aberration
	c.7828G>A (p.Val2610Met)	Donor 89.26 (+1.32%)	73.16	Donor 9.99 (+57.57%)	3.1	Donor 1.00	NSC	SRp55∶74.69 (+4.97%)	no aberration
	c.8734G>A (p.Ala2912Thr)	Acceptor 75.26 (+0.09%)	82.1	NSC	6.97	NSC	0.98	SF2/ASF (IgM-BRCA1): site broken SF2/ASF: site broken	no aberration

* Bracketed percentages refer to the difference between variant and wild-type scores as a proportion of the wild-type score. NSC, no sites created (no scores provided by bioinformatic program output). Positive values for HSF matrices and MaxEntScan represent an increased likelihood of creating a *de novo* site when compared with the wild-type sequence where the variant occurs. Negative values represent a decreased likelihood. Positive values for ESEfinder represent an increase in strength for the enhancer motif as a result of the variant. The proximal consensus site is taken as the donor or acceptor site of the exon in which the variant occurs. Variant scores for NNsplice are for splice sites created by the variant, except for *BRCA1*:c.4484G>C (p.Arg1495Thr) for which the variant score is for the consensus splice junction in the presence of the variant.

### mRNA Splicing Assays

For *BRCA1*:c.4484G>C(p.Arg1495Thr), a blood sample was taken from the variant carrier using an RNA stabilising, PaxGene tube and RNA extracted within 24 hrs using the PAXgene Blood RNA Kit (Qiagen, Doncaster, Victoria, Australia). A blood sample was collected from one female healthy control using the same sample collection and RNA extraction protocol, for comparison in the splicing assay. For the remaining variants assessed, culture of LCLs was conducted without and with cycloheximide, where treated LCLs were grown in the presence of cycloheximide (100 mg/ml) for 4 hours to stabilize transcripts against nonsense mediated decay (NMD) to assist detection of aberrant mRNA products [17]. RNA was extracted from cycloheximide untreated and treated cell lines using the RNeasy Mini Kit (Qiagen), according to the manufacturer’s instructions. Each RNA sample was treated with DNase to minimize DNA contamination using DNA-free Kit (Ambion, Austin, TX, USA). cDNA was synthesised using Superscript III First Strand Synthesis System (Invitrogen). PCR amplification was performed using Amplitaq Gold (Applied Biosystems, Mulgrave, Victoria, Australia) under the following conditions: 95°C for 7 minutes followed by 35 cycles of 94°C for 30 seconds, 55°C for 30 seconds and 72°C for 1–2 minutes and a final extension step at 72°C for 7 minutes (See Table S1 for details of primers). PCR products from the *BRCA1*:c.4484G> C(p.Arg1495Thr) carrier were purified using QIAquick PCR Purification Kit (Qiagen), cloned using pGEM-T Vector (Promega, Auburn, Victoria, Australia) and sequenced using Big-Dye Terminator version 3.1 sequencing chemistry and the ABI 377 sequencer (Applied Biosystems). The interpretation of the clinical significance of variants based on splicing data was as described in Walker et al [18].

### Multifactorial Likelihood Analysis

Data relevant for multifactorial analysis was available for all variants except *BRCA1*:c.4484G>C(p.Arg1495Thr). Information on segregation was available for all families, and likelihood ratios (LRs) based on tumor pathology (ER and grade for *BRCA1* and tubule formation for *BRCA2*) [19], co-occurrence and family history was available for a subset the variants (Table 2). Multifactorial analysis was conducted using the methods described in Walker et al [20] which incorporates likehoods for segregation [21], tumor pathology [21], [22], co-occurrence [23] and family history [24]. Family History and co-occurrence LRs were derived by querying a Myriad Genetics Laboratories dataset of 70,000 *BRCA1* and *BRCA2* tests [24]. However, in recognition of the recent report describing a patient with developmental delay and early onset ovarian cancer found to carry two pathogenic *BRCA1* mutations [25], for *BRCA1* variants we applied the same co-occurrence likelihood ratio formulation derived for *BRCA2* variants [8] which takes into consideration presentation of a Fanconi Anemia clinical phenotype in carriers of two pathogenic *BRCA2* mutations. Variant classifications follow the IARC criteria outlined in Plon et al. [3], namely: Class 1 not pathogenic posterior probability (pp)<0.001; class 2 likely not pathogenic pp 0.001–0.049; Class 3 uncertain pp 0.05–0.949; Class 4 likely pathogenic pp 0.95–0.99; Class 5 pathogenic pp>0.99. The classification system assigns recommendations related to surveillance and patient management guidelines [3].

**Table 2:** Classification of *BRCA1* and *BRCA2* variants on the basis of multifactorial and splicing information.

Variant		A-GVGD	A-GVGD prior probability	Segreg-ation	Tumor Patho-logy	Co-occurrence	Family History	Odds for Causality	Posterior Probability of Pathogenicity	IARC Class	Splicing class
***BRCA1***	c.122A>G(p.His41Arg)	C25	0.29	159.17	2.95	–	–	469.56	0.995	**Class 5**	–
	c.2759T>C (p.Val920Ala)	C0	0.01	0.002	–	–	–	0.002	1.52×10^−5^	Class 1	–
	c.4484G>C(Arg1495Thr)	C0	0.01	–	–	–	–	–	–	–	**Class 5**
	c.4991T>C (p.Leu1664Pro)	C0	0.01	0.01	–	1.29	0.03	0.0003	3.89×10^−6^	Class 1	Class 1
***BRCA2***	c.1354C>A (p.Leu452Ile)	C0	0.01	0.03	–	–	–	0.03	0.0003	Class 1	–
	c.440A>G (p.Gln147Arg)	C0	0.01	1.38	1.20	1.07	0.78	1.37	0.014	Class 2	Class 1
	c.1514T>C (p.Ile505Thr)	C0	0.01	0.16	1.20	–	–	0.191	0.002	Class 2	Class 1
	c.4609G>A (p.Glu1537Lys)	C0	0.01	0.004	–	–	–	0.004	4.24×10^−5^	Class 1	–
	c.5070A>C (p.Lys1690Asn)	C0	0.01	0.48	–	0.30	3.23×10^−5^	4.55×10^−6^	4.59×10^−8^	Class 1	–
	c.5278T>G (p.Ser1760Ala)	C0	0.01	1.38	0.14	–	–	0.1977	0.002	Class 2	–
	c.5714A>G (p.His1905Arg)	C0	0.01	0.16	–	–	–	0.16	0.0016	Class 2	–
	c.6172T>A (p.Phe2058Ile)	C15	0.29	0.04	–	1.12	0.20	0.008	0.003	Class 2	–
	c.6322C>T (p.Arg2108Cys)	C0	0.01	0.06	–	–	–	0.06	0.0006	Class 1	–
	c.7521A>G (p. = )	SYN	0.01	0.01	–	–	–	0.01	9.89×10^−5^	Class 1	Class 1
	c.7534C>T (p.Leu2512Phe)	C0	0.01	0.11	–	–	–	0.11	0.0011	Class 2	–
	c.7828G>A (p.Val2610Met)	C15	0.29	0.83	–	–	–	0.8288	0.25	Class 3	Class 1
	c.8734G>A (p.Ala2912Thr)	C0	0.01	0.14	–	–	–	0.14	0.0014	Class 2	Class 1
	c.9038C>T (p.Thr3013Ile)	C0	0.01	0.23	–	–	–	0.23	0.002	Class 2	–
	c.9364G>A (p.Ala3122Thr)	C0	0.01	0.34	–	–	–	0.34	0.0034	Class 2	–

Classifications for multifactorial likelihood as described in Plon et al. (3) and splicing as described in Spurdle et al. (32). Frequency data from 1000 Genomes and EVS datasets is available for a subset of the variants studied (Table S2). Information used to determine tumor pathology LRs was as follows: *BRCA1*c.122A>G(p.His41Arg) - one ER-positive Grade 3 tumor; *BRCA2* variants c.440A>G (p.Gln147Arg) and c.1514T>C (p.Ile505Thr) - tubule formation present in <10% of tumor; *BRCA2*:c.5278T>G (p.Ser1760Ala) – tubule formation in >75% tumor.

### Data Access

After peer-review and publication, the variants analysed and findings from this study will be submitted to several public databases: the LOVD literature unclassified variant database (http://chromium.liacs.nl/LOVD2/cancer/home.php?select_db=BRCA1); the LOVD-IARC ex-UV database (http://brca.iarc.fr/LOVD/home.php?select_db=BRCA1) which shows results from multifactorial likelihood analyses of BRCA variants; the BIC database (http://research.nhgri.nih.gov/bic/) which collates information from these databases and the scientific literature to derive a curated, publicly available classification source.

## Results

### Bioinformatic Analysis of Variants

HSF, MaxEntScan and NNsplice predicted a reduction in splice site strength at the intron-exon boundary for *BRCA1*:c.4484G> C(Arg1495Thr), which occurs in the last nucleotide of exon 14 and a strongly increased *de novo* splice site attributable to *BRCA2:*c.7828G>A(p.Val2610Met) (Table 1). Changes to normal splicing for the other 17 variants were very modest (<25% difference between wildtype and variant values) and/or variant score was considerably lower than expected for donor/acceptor sites at the consensus sequence at intron-exon boundaries. Of the variants investigated using mRNA assays (Table 1), ESEfinder predicted potential disruption of binding of splice regulatory proteins for *BRCA2*c.440A>G(p.Gln147Arg) and c.8734G>A (p.Ala2912Thr), and a new site or increase in binding for *BRCA2*c.440A>G(p.Gln147Arg) and three other variants.

### mRNA Analysis Reveals that *BRCA1*:c.4484G>C(Arg1495Thr) Produces Two Aberrant Transcripts with Whole Exon Deletions

**Figure 1:**
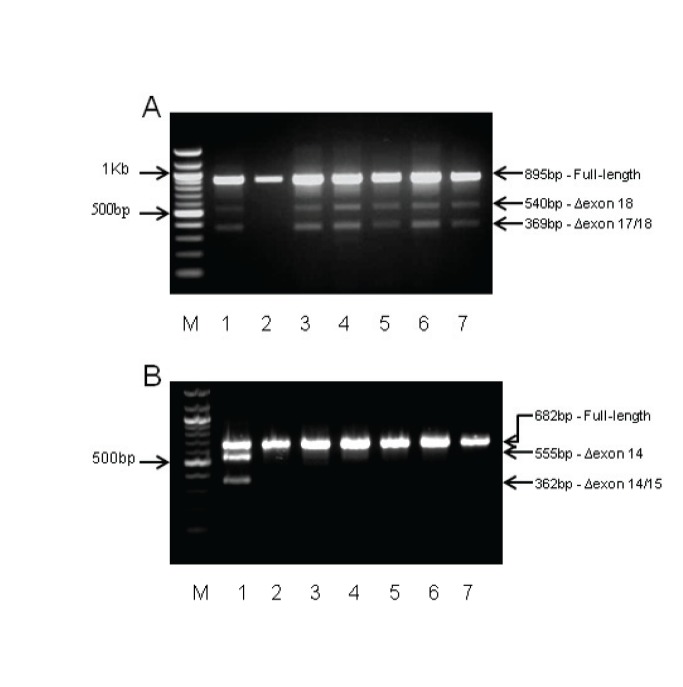
RT-PCR results for *BRCA1* c.4484G>C(p.Arg1495Thr) and *BRCA2:*c.7828G>A (p.Val2610Met). *M* - 100bp DNA marker (New England Biolabs). A) *BRCA2*:c.7828G>A (p.Val2610Met). Lane 1: RT-PCR products from variant carrier derived cycloheximide treated LCL. Lane 2–7: Cycloheximide treated LCLs from unaffected female controls. There is no evidence for a predicted loss of 149bp from exon 17 as a result of a *de novo* donor site. The Δexon 18 (540bp) and Δexon 17/18 (369bp) are detected in the variant carrier and all but one control samples. B) *BRCA1* c.4484G>C(p.Arg1495Thr). Lane 1: RT-PCR products from whole blood derived RNA from the variant carrier showing the Δexon 14 and Δexon 14/15 splicing aberration. Lane 2: RT-PCR carried out on whole blood derived RNA from an unaffected female control (collection and extraction methods as per the variant carrier). Lane 3–7: Cycloheximide treated LCLs from unaffected female controls.

As described in the methods, mRNA splicing assays were performed for a subset of 7 variants selected according to existing availability of material, and bioinformatic predictions that justify performing mRNA assays (Table 1). Despite predictions of an increased likelihood of creating a *de novo* donor site by MaxEntScan for *BRCA2:*c.7828G>A(p.Val2610Met), there was no evidence for the predicted aberration from RT-PCR analysis (Figure 1A).

Aberrant mRNA splicing was detected experimentally for the other variant for which there was a bioinformatic prediction of an effect on splicing: *BRCA1*: c.4484G>C(p.Arg1495Thr) variant located at the last base of exon 14. Consistent with the prediction by HSF and MaxEntScan of loss of a donor site, two aberrant splice products were detected by gel electrophoresis of RT-PCR products in the variant carrier but not in controls (Figure 1B). Sub-cloning and sequencing of the PCR products confirmed that the 555 bp splice product contains an out-of-frame deletion of exon 14 predicted to encode a truncating protein, although the presence of the stop codon will likely result in degradation by nonsense-mediated decay (NMD). The 362 bp product was shown to represent a transcript with an in-frame deletion of exon 14/15, covering the BRCA1 transactivation domain. The full-length product was extracted from the agarose gel following electrophoresis and sequenced. Only wild-type sequence was evident in the chromatogram data suggesting that the variant allele does not produce full-length transcript.

### Multifactorial Likelihood Analysis

As shown in Table 2, classifications after multifactorial analysis of the 18 of the 19 exonic variants assessed were: class 1 (not pathogenic) for 7 variants, class 2 (likely not pathogenic) for 9 variants, class 3 (uncertain) for 1 variant, and class 5 (Pathogenic) for 1 variant. The posterior probability of pathogenicity for *BRCA1*:c.122A>G(p.His41Arg) was 0.995, driven predominantly by a strong co-segregation score (159.17) calculation from two families. *BRCA1*:c.2759T>C(p.Val920Ala) and *BRCA2*:c.5278T>G(p.Ser1760Ala) were previously analyzed by multifactorial likelihood analysis and were determined to be Class 3 and Class 2 respectively, based on the data available at that time [22]. The Bayes odds for *BRCA1*:c.2759T>C (p.Val920Ala) was 0.9811 in the initial study [22] and genotyping of additional family members in this study lowered the Bayes segregation odds to 0.002, resulting in a revised posterior probability of 1.52×10^−5^ (Class 1). For *BRCA2*:c.5278T>G(p.Ser1760Ala), additional genotyping of six individuals changed the Bayes segregation odds from 1.17 in the initial study to 1.38 in this study but did not alter the Class 2 classification.

## Discussion

Results from this study provide evidence of pathogenicity for the two variants *BRCA1*:c.4484G>C(p.Arg1495Thr) and *BRCA1*:c.122A>G(p.His41Arg), and indicate that another 16 variants are not associated with high risk of cancer (Class 1 or 2). These findings are of direct relevance for counselling and management of individuals found to carry these variants. They also highlight the need for ongoing collection of clinical data to facilitate classification, as recommended for variants that fall into IARC Class 2, 3 or 4 [3]. Specifically, the inclusion of additional genotypes into the Bayes segregation analysis for *BRCA1*:c.2759T>C(p.Val920Ala) was a major factor which led to reclassification of this variant from Class 2 to Class 1.

The *BRCA2:*c.7828G>A(p.Val2610Met) variant which fell into Class 3 on the basis of multifactorial likelihood modelling was also investigated using mRNA assays since bioinformatic analysis using HSF, MaxEntScan and NNsplice predicted a splice donor. The lack of splicing aberration indicates the variant is in fact not deleterious due to effect on splicing. Given only modest likelihood of an effect on protein function (prior probability of 0.29 based on missense effect, conservation and location), further clinical information will be most helpful to resolve the clinical significance of this variant.

Bioinformatic splice prediction of *a de novo* donor for *BRCA2*:c.7828G>A(p.Val2610Met) was not confirmed experimentally, with no evidence observed for a splicing aberration caused by this variant. The sequence at the next downstream intron-exon boundary is CAG**gc**aagt, which contains a gc dinucleotide rarely observed at the intron-exon boundary. NNsplice did not predict a splice junction, and the score for the donor sequence at this motif was 3.1 for MaxEntScan and 73.16 for HSF. This compared to 9.99 (MaxEntScan) and 89.26 (HSF) for the *de novo* donor predicted for the variant. That is, the higher bioinformatic scores for the variant suggest that the *de novo* motif should out-compete the motif at the intron-exon boundary, and the normal splicing profile observed for this variant is thus surprising. This finding raises the possibility that splicing regulation in this region may be strongly dependent on *cis*-acting regulatory motifs and accessibility for the polypeptides and small nuclear RNAs that coordinate splicing [20], [26]. While we cannot exclude the possibility that the discrepancy between prediction and assay result reflects tissue-specific splicing events that are restricted to breast epithelium, there is much evidence demonstrating validity of mRNA assays using blood-derived tissue sources. Specifically, 12 of 13 reported naturally-occurring *BRCA1* splice variants detected in breast tissue also occur in lymphocytes [27], and we have recently demonstrated the validity of LCLs as a tissue source for routine mRNA assays of gene variants leading to major aberrations [28].

The *BRCA1* c.4484G>C(p.Arg1495Thr) variant was shown by our analysis to create aberrant splice products encoding loss-of-function proteins. This variant is located at the last base of exon 14, with increased bioinformatic likelihood to disrupt normal donor function. The exon 14 splicing defect observed in mRNA analysis of *BRCA1*c.4484G>C(p.Arg1495Thr) was also previously reported for a different variant (*BRCA1*:c.4484G>T) at the same nucleotide [29], [30]. While RT-PCR is not quantitative and may not reflect the true ratio of full-length to aberrant transcript, the variant allele appears to produce only exon 14 deletion and exon 14/15 deletion transcripts. The exon 14 deletion leads to an out-of-frame transcript, so impact on the protein can be unambiguously inferred from the sequence information. Interpreting the effect of the exon 14/15 in-frame deletion on protein function is not as simple. This deletion falls within a broad TAD region c.3879 to c.5592 defined by BRCA interaction with transcription partners LMO4, JunB and HDACs [31]–[33], but does not incorporate the BRCT regions essential for TAD function [34] and might thus be considered to lie within auxillary activating regions. Further, while there is an example of a variant in exon 13 (BRCA1 p.Leu1407Pro) resulting in loss of transactivation activity [35], the exon14/15 deletion itself would appear to be a poor candidate for loss of transactivation function: it is not well conserved evolutionarily, it is not predicted bioinformatically to alter stability (data not shown), and to our knowledge has not been tested for effect on TAD activity. Nevertheless, the *BRCA1* del exon14–15 splicing variant has been demonstrated to impair DNA double-strand break repair and also to interfere with the activity of wildtype BRCA1 in a dominant-negative fashion [36], via loss of non-homologous end-joining activity. Thus, it is appropriate to consider that both aberrant transcripts caused by the *BRCA1*:c.4484G>T substitution are deleterious to protein function, and to place this variant in Class 5 on the basis of the mRNA assay data. Although, the exercise of interpreting the functional importance of the exon14/15 deletion has highlighted the need to standardize definitions of functional domains in *BRCA1* or *BRCA2* that consider differences in effects of missense versus in-frame deletions, this interpretation as Class 5 is consistent with the IARC Unclassified Genetic Variants Working Group recommendations [37] recently revised for clarity [18], namely: “variant allele produces only transcript(s) carrying a premature stop codon or an in-frame deletion disrupting known functional domain(s)”.

Together, the results from our mRNA assays highlight the importance of considering potential splice defects for exonic variants, but also the need to improve bioinformatic prediction tools by incorporating information about other motifs and factors important for splicing. The data presented in this study will add to a pool of information that may be used, in the future, to calibrate bioinformatic predictions and/or splicing assay results against cancer risk as measured using clinical data. Such calibration is important, since although mRNA assays are commonly used in clinical testing to detect splicing aberrations and infer pathogenicity, the interpretation of assay data is challenging where the variant allele produces multiple transcripts e.g. a combination of full-length, naturally occurring isoforms, and aberrant transcripts. The ENIGMA Splicing Working group has highlighted the need to move to quantitative assays for future calibration analyses [18], and it is encouraging that appropriate technologies are becoming available, including a pyrosequencing approach allowing accurate measure allelic ratios of splice isoforms in patient RNA [38].

While there are a range of functional assays used to elucidate protein interactions and cellular mechanisms affected by *BRCA1* and *BRCA2* missense variants, incorporation of functional assay data in the model is at present limited. Firstly, the execution and interpretation of such results is generally limited to specialists in the field. Secondly, functional assays are not a direct measure of cancer risk, and therefore need to be calibrated for sensitivity and specificity against appropriate variants of known clinical significance i.e. “high-risk” pathogenic or clearly not pathogenic variants, located in domains relevant to the functional assay being assessed. The *BRCA1*:c.122A>G(p.His41Arg) variant classified here as pathogenic by multifactorial analysis is located in the RING domain. The RING-domain spans amino acids 8–96, and includes a binding site for the BARD1 protein [39], which in turn enables the E3 ubiquitin ligase activity of BRCA1 observed at sites of DNA repair [40], [41]. Further, structural changes to the protein involving co-ordination of zinc ions can result in loss of homologous recombination activity [42], [43]. Results from our study add to the body of evidence on the relationship between loss of each of these functions and cancer risk.

As summarized in Table 3, there are 26 variants in the BRCA1 RING-domain that have been tested for BARD1 binding activity and Ubiquitin ligase activity and/or, classified using the multifactorial approach. Several of these variants have also been assessed for loss of function using a homology-directed recombination assay. The p.His41Arg protein has been reported to show weak BARD1 binding capacity and abrogated E3 ubiquitin ligase activity [43]. Considering the remaining 20 variants assessed for BARD1 binding and ubiquitin ligase function, results reported indicate that 6 variants lost both BARD1 binding capacity and ubiquitin ligase activity, 12 variants lost ubiquitin ligase activity with no or inconclusive BARD1 binding (2 variants), one variant lost only BARD1 binding capacity, and another exhibited inconclusive ligase activity.

**Table 3:** BRCA1 RING-domain variants with reported loss of function on the basis of *in-vitro* functional assays and/or (likely) clinically significant from multifactorial likelihood analysis.

BRCA1 Mutation	BARD1Binding	UbiquitinLigase Activity	HomologyDirected Repair	Posteriorprobability	Classification	Ref.
p.Val11Ala	Lost	Retained	–			[46]
p.Ile15Thr	Retained	Abrogated	–			[43]
p.Met18Lys	Lost	Abrogated	–			[46]
p.Met18Thr*	Retained	Abrogated	Abrogated	0.984	Class 4- Likely pathogenic	[24], [42], [43], [46]
p.Leu22Ser	–	–	–	0.994	Class 5 - Pathogenic	[47]
p.Cys24Arg	Lost	Abrogated	Abrogated			[42], [43], [48]
p.Ile26Ala	Lost	Abrogated	–			[43]
p.Leu28Pro	Retained	Inconclusive	–			[43]
p.Thr37Arg	Lost	Abrogated	Abrogated			[42], [43], [48]
p.Thr37Lys	–	–	–	0.999	Class 5 - Pathogenic	[47]
p.Cys39Arg	Retained	Abrogated	–	0.993	Class 5 - Pathogenic	[43], [47]
p.Cys39Tyr	Lost	–	–			[42]
p.His41Arg	Weak Binding	Abrogated	Abrogated	0.995	Class 5 - Pathogenic	[42], [43], present study
p.Cys44Phe	Lost	Abrogated	Abrogated			[42], [43]
p.Cys44Ser	–	–	–	0.998	Class 5 - Pathogenic	[47]
p.Cys44Tyr	–	–	–	0.997	Class 5 - Pathogenic	[47]
p.Lys45Thr	Retained	Abrogated	–			[43]
p.Lys45Asn	Retained	Abrogated	–			[43]
p.Cys47Gly	Retained	Abrogated	Abrogated			[42], [43]
p.Leu52Phe	Inconclusive	Abrogated	No impact			[42], [43],
p.Cys61Gly*	Lost	Abrogated	Abrogated	0.999	Class 5 - Pathogenic	[42], [43], [47]
p.Leu63Phe	Retained	Abrogated	–			[43]
p.Cys64Gly*	Inconclusive	Abrogated	Abrogated			[42], [43]
p.Ile68Lys	Retained	Abrogated	–			[43]
p.Ser72Arg	Retained	Abrogated	–			[43]
p.Thr77Met	Retained	Abrogated	–			[43]

* p.Met18Thr, p.Cys61Gly and p.Cys64Gly are also shown to have abrogated function using mouse embryonic stem cell assays [44], [49]. (No other variants listed in Table 3 were assayed using this method).

In all eight instances of abrogated homology directed repair function, abrogated ubiquitin ligase activity function by was also observed, although the reverse is not always the case. For example, there is one variant (p.Leu52Phe) that despite abrogated function by ubiquitin ligase activity had no impact on function in the homology directed repair assay. Of the eight variants, where both ubiquitin ligase activity and homology directed repair showed abrogated function, five also lost or had weak BARD1 binding, one showed inconclusive binding and two retained BARD1 binding (p.Met18Thr and p.Cys47Gly). Taken together, this indicates that different variants may have different effects on the function of the RING domain, and that no single assay should be used to infer loss of function at this point in time. Alternatively, it may be preferable to consider as an alternative assays such as the mouse embryonic stem cell assay [44] with cell proliferation as outcome measure, which can be used to indirectly measure the functional capacity of variants in the RING finger and also other protein domains.

Comparing functional assay results with clinical classification of pathogenicity variants using multifactorial analysis (Class 4 and 5), four variants show variously: abrogated ubiquitin ligase and retained BARD1 binding (p.Met18Thr and p.Cys39Arg); abrogated ubiquitin ligase and weak BARD1 binding activity (p.His41Arg); abrogated ubiquitin ligase activity and lost BARD1 binding (p.Cys61Gly). Each of these four variants also showed abrogated function in the homology directed repair assay except p.Cys39Arg which remains untested. These observations are notable, given the conclusion by Shakya et al. that E3 ubiquitin ligase activity is not required for tumor suppression [45]. Of the remaining variants classified using the multifactorial approach, all Class 5, none have been tested in functional assays. It would be of interest, in order to improve our understanding of the relationship of function to risk, for further studies to assess RING domain functions for these 4 variants, and yet other studies to determine the clinical significance using multifactorial analysis for the 17 variants with existing functional assay data. Such studies will pave the way to incorporation of assays measuring the various functions of the RING finger domain into future multifactorial models.

In summary, our investigations have provided information of clinical utility for 18 of 19 *BRCA1* or *BRCA2* variants identified by clinical germline testing of breast cancer patients. Our results also provide further evidence that bioinformatic predictions of altered splicing should be incorporated into clinical assessment of variants to prioritize mRNA assays, and used to improve bioinformatic splicing prediction tools and the estimation of the prior probability of pathogenicity for assumed missense alterations. Lastly, the classifications arising from our study will be useful for future studies that correlate functional or splicing assay results against risk.

## Supporting Information

Table S1 Primers for mRNA splicing assays.
(DOCX)

Table S2 Frequency of variant occurrence in 1000 Genomes and EVS snp datasets.
(DOCX)
