# Supplementary material for: Multifactorial Likelihood Assessment of BRCA1 and BRCA2 Missense Variants Confirms That BRCA1:c.122A>G(p.His41Arg) Is a Pathogenic Mutation
Source: PLoS One. 2014 Jan 28;9(1):e86836. doi: 10.1371/journal.pone.0086836 (PMC3904950; doi:10.1371/journal.pone.0086836)
Supplement: Table S1 — Primers for mRNA splicing assays. (DOCX) [file pone.0086836.s001.docx]

**Supplementary Table S1. Primers for mRNA splicing assays**

| Variant | Exon containing variant | Forward primer 5’ to 3’ | Exon containing primer | Reverse Primer 5’ to 3’ | Exon containing primer |
| --- | --- | --- | --- | --- | --- |
| *BRCA1*:c.4484G>C(Arg1495Thr) | 14 | CAGCAGGAAATGGCTGAACT | 13 | CCCTGCTCACACTTTCTTCC | 16 |
| BRCA1:c.4991T>C (p.Leu1664Pro) | 17 | GTCATCCCCTTCTAAATGCC | 15 | GGCTCTGTACCTGTGGCTGGC | 24 |
| BRCA2:c.1514T>C (p.Ile505Thr) | 10 | CAAAGAGAAGCTGCAAGTCA | 9 | TTCTTTTCACAGAAGAATGCAA | 11 |
| BRCA2:c.440A>G (p.Gln147Arg) | 5 | GCAGATGATGTTTCCTGTCC | 4 | TGACTTGCAGCTTCTCTTTGA | 9 |
| BRCA2:c.7521A>G (p.=) | 15 | AAAAGATCGAAGATTGTTTATGC | 13 | CCATCAGCCAACTGTATTCC | 16 |
| BRCA2:c.8734G>A (p.Ala2912Thr) | 21 | GAAGCCCCAGAATCTCTTATG | 18 | CTCTTAACTGCTCTTCACTG | 22 |
| *BRCA2:*c.7828G>A (p.Val2610Met) | 17 | CAGCCAGGCAGTCTGTATCT | 15 | GCAGAGGAAAAGGTCTAGGG | 19 |
