# Supplementary material for: Multifactorial Likelihood Assessment of BRCA1 and BRCA2 Missense Variants Confirms That BRCA1:c.122A>G(p.His41Arg) Is a Pathogenic Mutation
Source: PLoS One. 2014 Jan 28;9(1):e86836. doi: 10.1371/journal.pone.0086836 (PMC3904950; doi:10.1371/journal.pone.0086836)
Supplement: Table S2 — Frequency of variant occurrence in 1000 Genomes and EVS snp datasets. (DOCX) [file pone.0086836.s002.docx]

|  |  | 1000 Genomes | | | | EVS | |
| --- | --- | --- | --- | --- | --- | --- | --- |
|  | variant | Caucasian | Admixed Americans | Asian | African | Asian | African |
| BRCA1 | c.122A>G |  |  |  |  |  |  |
| BRCA1 | c.2759T>C |  |  |  |  |  |  |
| BRCA1 | c.4484G>C |  |  |  |  |  |  |
| BRCA1 | c.4991T>C |  |  |  |  |  |  |
| BRCA2 | c.1354C>A |  |  |  |  |  |  |
| BRCA2 | c.440A>G |  |  |  |  |  |  |
| BRCA2 | c.1514T>C | 0.002639 | 0 | 0 | 0 | 0.001047 | 0.000227 |
| BRCA2 | c.4609G>A |  |  |  |  |  |  |
| BRCA2 | c.5070A>C |  |  |  |  | 0.000233 | 0 |
| BRCA2 | c.5278T>G |  |  |  |  |  |  |
| BRCA2 | c.5714A>G |  |  |  |  |  |  |
| BRCA2 | c.6172T>A |  |  |  |  |  |  |
| BRCA2 | c.6322C>T | 0.002639 | 0 | 0.005245 | 0 | 0.000465 | 0 |
| BRCA2 | c.7521A>G |  |  |  |  |  |  |
| BRCA2 | c.7534C>T |  |  |  |  | 0.000116 | 0 |
| BRCA2 | c.7828G>A |  |  |  |  |  |  |
| BRCA2 | c.8734G>A |  |  |  |  |  |  |
| BRCA2 | c.9038C>T |  |  |  |  | 0.000582 | 0.000227 |
| BRCA2 | c.9364G>A |  |  |  |  |  |  |

**Supplementary Table S2. Frequency of variant occurrence in 1000 Genomes and EVS snp datasets.**
